# Access Control Based on Ciphertext Attribute Authentication and Threshold Policy for the Internet of Things

**DOI:** 10.3390/s19235237

**Published:** 2019-11-28

**Authors:** Qikun Zhang, Yongjiao Li, Zhigang Li, Junling Yuan, Yong Gan, Xiangyang Luo

**Affiliations:** 1 School of Computer and Communication Engineering, Zhengzhou University of Light Industry, Zhengzhou 450002, China; zhangqikun04@163.com (Q.Z.); lizg.cn@hotmail.com (Z.L.); yuanjunling@foxmail.com (J.Y.); 2 Zhengzhou Institute of Technology, Zhengzhou 450044, China; ganyong@zzuli.edu.cn; 3 State Key Laboratory of Mathematical Engineering and Advanced Computing, Zhengzhou 450002, China; xiangyangluo@126.com

**Keywords:** Internet of Things, attribute-based encryption, access control, data security sharing, attribute authentication

## Abstract

The development of the Internet of Things has led to great development of data sharing and data interaction, which has made security and privacy more and more a concern for users. How to ensure the safe sharing of data, avoid the leakage of sensitive information, and protect the privacy of users is a serious challenge. Access control is an important issue to ensure the trust of the Internet of Things. This paper proposes an access control scheme based on ciphertext attribute authentication and threshold policy, which uses the identity authentication of hidden attributes and divides the user’s permission grade by setting the threshold function with the user’s attributes. Users obtain different permission grades according to attribute authentication and access data of different sensitivity grades to achieve fine-grained, flexible and secure access to data in the cloud server while protecting personal privacy issues. In addition, when the resource is acquired, the identity and permission joint authentication method is adopted to avoid the collusion attack of the illegal member, which makes the resource access control more secure.

## 1. Introduction

The development of the Internet of Things has spawned the emergence of new informatization concepts such as smart homes, smart cities, and mobile crowd sensing. It connects people, people and things, things and things on the Internet, and realizes information exchange, collaborative operation, and resource sharing among terminal entities through wired or wireless network technologies according to different physical environments and application scenarios [1]. It is widely used in various fields of the information society, such as remote control of intelligent terminals for remote medical treatment, unmanned vehicle driving in the in-vehicle networks and intelligent sensor data, and remote physical environment monitoring in remote areas. The development of the Internet of Things has brought great convenience to people’s lives, promoted the rise of the sharing economy, and promoted the development of society.

At the same time, the development of the Internet of Things is also facing some serious challenges: In the field of physical technology, with the improvement of people’s living standards, people’s application requirements for the Internet of Things are constantly improving, such as the real-time nature of Internet of Things communication, the bandwidth of the Internet of Things, and the energy consumption of the Internet of Things. Different application scenarios will also involve specific requirements regarding scope, power consumption, throughput, and network topology. These are issues that the Internet of Things needs to further improve and solve [2]. In the field of information application, the popularization and application of the Internet of Things involves various fields of people’s life and work. The vulnerability of the Internet of Things makes the communication information, identity information, and shared information among terminal entities easily exposed to the open Internet of Things. Securing the confidentiality of IoT communication information, the concealment of identity information, and the security of shared information are another challenge facing the Internet of Things. Therefore, it is necessary to develop the innovative information technology of the Internet of Things, the privacy protection of users, and the security of interactive data and shared data [3].

The powerful interoperability of the Internet of Things has accelerated the data aggregation and data sharing in the Internet of Things, making the Internet of Things one of the infrastructures in many fields such as medical and intelligent transportation. To protect data security in the Internet of Things, data are typically stored in encrypted form on the server, and attribute-based encryption has proven to be a powerful encryption tool. At the same time, it is crucial to propose effective access control policies to access these encrypted data and protect the security of some sensitive information. In recent years, research on access control of data has emerged in an endless stream attribute-based encryption strategies are widely used in medical, corporate, and personal areas. Privacy protection and field access for personal health information forms for medical use attribute-based encryption methods, and permission settings for access control [4], which can protect privacy and flexible access, bringing great benefits to the medical industry. A comprehensive study of some different access control models and access control architectures provides a future direction for IoT access control research [5]. Verifiable threshold multi-permission access control constructs a mixed multi-permission scheme [6]; on the basis of one permission maintaining the whole attribute set encryption scheme, unified attribute setting managed by multiple departments can guarantee the security of data.

With the rapid development of big data, security in the fields of information storage, information acquisition, and information transmission has become a serious problem. The powerful interoperability and flexible access features of the Internet of Things have greatly threatened data security and personal privacy. Aiming at these problems, this paper proposes an access control scheme based on ciphertext attribute authentication and threshold policy for the Internet of Things (AC-CAATP), which uploads the data to the cloud server after encrypting the data, and uses attributes to set a threshold policy to limit the user’s access to the data. Similarly, the user authenticates the identity based on the attribute and obtains different permission grades from the certificate authority, and then accesses the data of the corresponding sensitivity grade. The protocol combines the advantages of attribute-based encryption technology and access control strategy to ensure data security and user privacy. At the same time, an access control policy is set for data access, which avoids the leakage of sensitive information.

### 1.1. Contributions

In this paper, an access control scheme based on ciphertext attribute authentication and threshold policy for the Internet of Things is proposed. The research contributions of this paper can be summarized as follows:

(1) Hidden attribute authentication: An identity authentication technology based on hidden attributes is proposed, which not only hides the user’s identity information, but also hides the user’s attribute information. In the process of identity authentication, the advantages of the traditional key agreement protocol identity authentication are preserved, and the attribute information is hidden by the algorithm to avoid revealing the user’s personal privacy.
(2) Lightweight and efficient access control technology: Traditional attribute-based access control mostly adopts complex access control policies, such as tree-based access control policies, which require a large number of intermediate nodes to calculate and transmit messages during data access, thus increasing a large amount of computational and communication overhead. In this paper, permission list query and threshold function are used to implement access control. A large number of nodes are not required to transmit information during the access process. Therefore, the computational amount is small and the computational time is short, which is more suitable for a mobile terminal device with limited resources and requiring fast resource access.
(3) High security: Traditional attribute-based access control is difficult for resisting collusion attacks. For example, when a user does not have enough attribute sets to access a resource, he can get enough attributes in conjunction with other users who do not have access to the resource to gain access to the resource. In this paper, the identity and permission joint authentication method is adopted in resource acquisition. When each user registers, the same attribute of different users obtains different permission parameters. Therefore, users cannot jointly access resources by using other members’ attributes. At the same time, this paper also adopts permission authentication when accessing resources. Users who do not reach the access level cannot access and download ciphertext resources, making resource access more secure.

### 1.2. Organization

In Section 2, the related work of this paper is described; in Section 3, the basic knowledge is described; in Section 4, the details of AC-CAATP scheme are described; in Section 5, the correctness and security analysis of AC-CAATP is described, and we further analyze the efficiency of AC-CAATP in Section 6; the conclusions of the paper are in Section 7.

## 2. Related Work

While the Internet of Things is widely used, the vulnerability of its network is subject to severe security challenges. In recent years, IoT security has become a personal issue for scholars at home and abroad, especially IoT data security exchange, IoT intrusion detection, and IoT access control. Intrusion detection research based on the Internet of Things was proposed in [7,8,9]; three methods are proposed in the literature to detect intrusion events in the network, which can prevent and resolve malicious attacks and improve the security of IoT applications. An overview of secure communication among vehicles is presented in [10], which describes cases in which multiple connected entities interact with appropriate communication protocols, analyzing, researching, and evaluating the most relevant systems, applications, and communication protocols, further improving road safety and predicting the potential danger of road traffic. In [11], a data exchange scheme based on wireless devices in a physical Internet of Things is proposed. It is based on two core elements with interchangeable roles, entities and trackers, using a blockchain-based distributed paradigm, existing infrastructure, and equipment to ensure anonymization and immutability of the data involved. This section focuses on the introduction and analysis of research work related to IoT access control.

In this era of information explosion and network sharing, safeguarding data and information security is a serious challenge for us. Some access to sensitive information requires some permission. More and more scholars have analyzed and studied this situation. In [12], a novel attribute based access control scheme for IoT systems is proposed, which simplifies greatly the access management. It uses block-chain technology to record the distribution of attributes in order to avoid single point failure and data tampering. The access control process has also been optimized to meet the need for high efficiency and lightweight calculation for IoT devices. In [13], the authors comprehensively expound the existing access control mechanisms used in the cloud computing environment, analyze the advantages and disadvantages of these models and application requirements, and evaluate the existing access control mechanisms based on these requirements.

In [14,15], in view of the limitation of medical resources in the Internet of Things, an access control system is proposed. The system provides authorized users with fine-grained access to services while protecting valuable resources from unauthorized access. In addition, the application attribute assigns a role to the member and authorizes the member to access the IoT device to provide a specific service. A secure and efficient multi-authority access control system for IoT-enabled mHealth is proposed in [16], there are multiple independent attribute authorities in the system, and a new entity can attribute authority without rebuilding the system. In addition, most of the decryption is performed in the cloud server, only returning part of the decrypted ciphertext, greatly reducing the user’s decryption overhead.

In [17], a scalable enhanced key aggregation cryptosystem is proposed to implement security level management. This method uses the improved Diffie–Hellman key exchange algorithm (IDHKE) to achieve secure data sharing and key security sharing of data receivers. For security and consistent access control restrictions, attribute-based encryption is used to ensure the accuracy and reliability of protected data transmission. In [18,19], the medical big data security utilization encryption and access control process was analyzed. The classification method is used to classify and encrypt sensitive data and non-sensitive data in cloud computing, and triple DES (TDES) is encrypted and stored in the cloud, and a feasible optimization technique is proposed, Finally, the attribute-based access control authentication phase is used to authenticate the data in the cloud sim. The clustering, classification and encryption results of the method are compared with existing methods.

In order to protect the confidentiality of data and solve the problem of leaking data or leaking user keys for different reasons based on attribute conditions, an attribute-based batch cloud access control system is proposed in [20] that introduces an efficient, revocable, attribute-based encryption scheme that enables data owners to efficiently manage the credentials of data users. In [21], a new security and privacy-based Connected Vehicle Network Access Control (SPBAC) model is proposed, which allows security officials to access information through permissions and roles, rather than just accessing the same fleet of officials through roles. The model sets up multiple security layer maintenance, and each security layer coordinates and communicates with each other to avoid leakage of sensitive information.

A security solution based on RFID card-based physical biometric access control is proposed in [22]. This scheme combines RFID technology and dual watermark technology to provide a biometric control access framework. The wavelet packet decomposition watermark algorithm is used to insert fingerprint (detail) features. Into the face image of the authorized person, the same watermark algorithm is then used to insert the fingerprint watermark into the face feature extracted by the Gabor filter in the previously watermarked face image, and, finally, the obtained secure watermark biometric data are integrated into the RFID card, efficiently preventing information theft and illegal access to sensitive data. In [23], the paper analyzes the key indicators affecting the privacy disclosure of big data in health management, and establishes a risk-based access control model based on fuzzy theory for intelligent medical big data management, which solves the problem that the experimental results are inaccurate due to the lack of real data when the actual problem is processed. Protecting information security issues in smart medical management largely avoids patient privacy issues.

A security model based on IoT and fog cooperation is proposed in [24]. The model integrates an efficient access control process associated with the monitoring solution to ensure secure cooperation between different resources and different operational parts. Introduce a distributed access control based on the secure resource management framework for the fog-IoT network, and an active security scheme under super-trusted and low latency constraints. The solution not only has low latency, high security and confidentiality, but also reduces the management and management complexity of security and resource mechanisms. A distributed access control with outsourced encryption and decryption for electronic health records is introduced in [25]. The device combines the advantages of the fog device, which provides calculation, transmission, and storage services for the user, so the communication and calculation costs are lower. This scheme is a practical and novel solution.

In [26,27], in order to implement a secure service composition, a privacy-protected access control model and framework is proposed. In the model, an access request for a service is permitted if the requester’s attribute certificates and contextual conditions are in compliance with the access control policies specified by the service provider and simultaneously the privacy preferences of the requester are compatible with the privacy policies of the service provider. In the framework, the possible combined service chains are sorted according to the user’s preference and the sensitivity of the data, and the security policy of the combined service is established by the selected service chain. A sensor platform for controlling an obstacle at the entrance of a vehicle is proposed in [28]. The platform enables automatic identification of the vehicle by image-based license plate recognition of the vehicle. First, an approaching vehicle is detected by an ultrasonic sensor, and, at the same time, an image is captured by a camera mounted on an obstacle, and then the license plate is automatically extracted from the image, and the license plate character is further divided. Finally, these characters are identified using standard optical character recognition (OCR) pipelines.

An access control system defined by blockchain technology is proposed in [29]. The system encodes the attribute-based access control policy into a smart contract and deploys it on the blockchain, thus transforming the policy evaluation process into a fully distributed intelligent contract execution, while the invariance and transparency of blockchain technology ensure the auditability of access control strategy evaluation. A new verifiable outsourcing ciphertext-policy attribute-based encryption scheme for big data privacy and access control in the cloud is proposed in [30,31], The solution reduces the computational overhead of encryption and decryption by outsourcing heavy computing to a proxy server, and verifies the correctness of the data through outsourced computation. In addition, the solution protects data security by limiting the data access of a group of users rather than providing unlimited data access.

In [32], cloud-based e-learning is implemented using an access control mechanism to prevent cloud resources from being accessed by unauthorized users. The system uses a key management scheme of access control technology to achieve secure content sharing and protection of the e-learning environment, and is more flexible and scalable in accessing e-learning content. A cloud computing data protection model is proposed in [33], which uses cryptography and access control to ensure the confidentiality, integrity, and proper control of sensitive data access. The model uses an enhanced RSA encryption algorithm combined with a role-based access control model and Extensible Access Control Markup Language (XACML) to improve security and allow data access. An edge-based encryption-based access control method (eri-ac) is proposed in [34]. This method encrypts the content using a symmetric key. The content key is secondarily encrypted by the producer and the edge router using edge re-encryption. Only authorized users can decrypt the re-encrypted content key with their private key to obtain the plaintext of the content. This method allows the user to obtain the content key from the producer, which can shorten the retrieval time and there is no copy redundancy.

In [35], the priority of the sensor is set according to the importance level of the sensor, the sampling rate, the timeout condition, and the remaining energy. Then, based on the priority of the node and the channel factor, a utility function is introduced to characterize the value of the node transmitting the data frame during a certain period of time. The time slot allocation problem is modeled. The goal is to maximize the total data transmission utility of all nodes in a specified time period by adjusting the transmission time and transmission duration of each node. According to the problem model, a time slot allocation scheme based on greedy strategy is proposed, which effectively reduces the time complexity of direct problem solving. In this scheme, nodes with higher priority are arranged to transmit data frames in time slots with better channel conditions. A transportation system boarding scheme for automatically controlling the number of passengers in the transport warehouse is proposed in [36]. The scheme uses the queuing theory to derive random numbers of passenger queue length, waiting time, and cabin capacity for determining the number of passengers arriving and the number of cabins arriving at Poisson. The expression of the nature deduces the cabin capacity and stability threshold of each station in the case of general passenger arrival distribution to control the number of passengers in the transport warehouse.

A crowdsourcing method for location-aware secure access (LaSa) control is proposed in [37], in which LaSa detects whether a user enters or leaves a room by discovering and identifying unique signal patterns. Combined with received signal strength (RSS), channel state information (CSI) and coarse angle of arrival (AoA) data, the accuracy of wireless network user classification is improved. In [38], a model is designed and presented in this study in order to enable the privileged accounts to be controlled, managed, and followed at minimum cost. The model can set a strong password based on basic IT security principles and refine the scope of IT staff to reduce their workload, and improve managers’ awareness of IT security to determine the password for a privileged user account. A function-based identity-based encryption-based IoT access control scheme is proposed in [39]. The program provides fine-grained access control to prevent applications from accessing unauthorized functions. At the same time, the cost of each access operation is a constant. In addition, the solution is secure, and prevents excessive privileged access.

At the same time, there are some studies on the analysis and evaluation of IoT security. A network and physical security vulnerability assessment based on the Internet of Things is proposed in [40], which outlines the application of the Internet of Things in the smart home sector, brings convenience and presents security and privacy challenges. Detect and identify possible security risks and vulnerabilities, fully understand the security status of smart homes, and propose ways to reduce risk. A survey of potential security issues with network protocols was proposed in [41]. The investigation raised the security and privacy issues in network protocols, analyzed vulnerabilities and security threats that are prone to networks, and agreed on new defense benchmarks.

It can be seen from the analysis of the above research results that the above research has a certain degree of deficiencies in terms of personal privacy protection, lightweight quality, and security. The rapid development of the Internet of Things has gradually changed people’s way of life, flooding all aspects of life, and people’s security requirements for the Internet of Things are getting higher and higher. According to the characteristics of the Internet of Things, such as limited mobile resources and easy disclosure of personal privacy, we propose an access control scheme based on ciphertext attribute authentication and threshold policy, in which further optimizations have been made in terms of personal privacy protection, lightweight and security. Through comparative analysis, the effect of this scheme is better.

## 3. Basic Knowledge and Security Assumptions

### 3.1. Bilinear Mapping

This paper is based on the basic theory of bilinear mapping; some basic knowledge related to bilinear mapping will be described in this section.

Let $G_{1}$ be an additive group and $G_{2}$ is a multiplicative group. Both of them have the same prime order *q*, where $q\geq 2^{\ell }+1$, and *ℓ* is a security parameter. $G_{1}$ is generated by $g_{1}$ that means $G_{1}=\langle g_{1}\rangle$, and the discrete logarithm problems of $G_{1}$ and $G_{2}$ are difficult. We call *e* an admissible pairing, if $e:G_{1}\times G_{1}\to G_{2}$ satisfies the follow properties:

(1) Bilinear: For all $u,v\in G_{1}$, and $a,b\in Z_{q}^{*}$, there is $e\left(au,bv\right)=e{\left(u,v\right)}^{ab}$;
(2) Non-degeneracy: There exists $u,v\in G_{1}$, such that e(v,u)≠1;
(3) Computability: For all $u,v\in G_{1}$, there exists a efficient way to calculate e(v,u).
  **Inference 1.** For all $u_{1},u_{2},v\in G_{1}$, there is $e\left(u_{1}+u_{2},v\right)=e\left(u_{1},v\right)e\left(u_{2},v\right)$.

### 3.2. Computational Complexity Problems

**Definition** **1.** *Discrete Logarithm problem (DLP). Given an equation Y=aP, where $Y,P\in G_{1}$ and a<q. If a and P are given, it is easy to calculate Y. However, if P and Y are given, it will be difficult to calculate a.*

**Definition** **2.** Decisional Bilinear Diffie–Hellman (DBDH) Problem. Assume $G_{1}=\langle g_{1}\rangle$ is an additive group and $G_{2}=\langle g_{1},g_{1}\rangle$ is a multiplicative group, Both of the two groups have the same large prime order q, where $q\geq 2^{\ell }+1$, and ℓ is a security parameter, $g_{1}$ is the generator of group $G_{1}$. $G_{1}$ and $G_{2}$ is a pair of bilinear group, and $e:G_{1}\times G_{1}\to G_{2}$ is a calculable bilinear mapping. The following two triples $\left(g_{1},g_{2},ag_{1},bg_{1},cg_{1},e{\left(g_{1},g_{1}\right)}^{abc}\right)$ and $\left(g_{1},g_{2},ag_{1},bg_{1},cg_{1},\pi \right)$, for any $a,b,c\in Z_{q}^{*}$, $g_{1}\in G_{1}$, $g_{2}\in G_{2}$ and $\pi \in G_{2}$ are computationally indistinguishable.

$KeyGen\left(1^{\lambda }\right)\to \left(sk,pk\right)$: It takes as inputs the security parameter λ, and outputs a public/private key pair (sk,pk).

## 4. The Proposed Access Control Scheme

### 4.1. System Model

**Figure 1:**
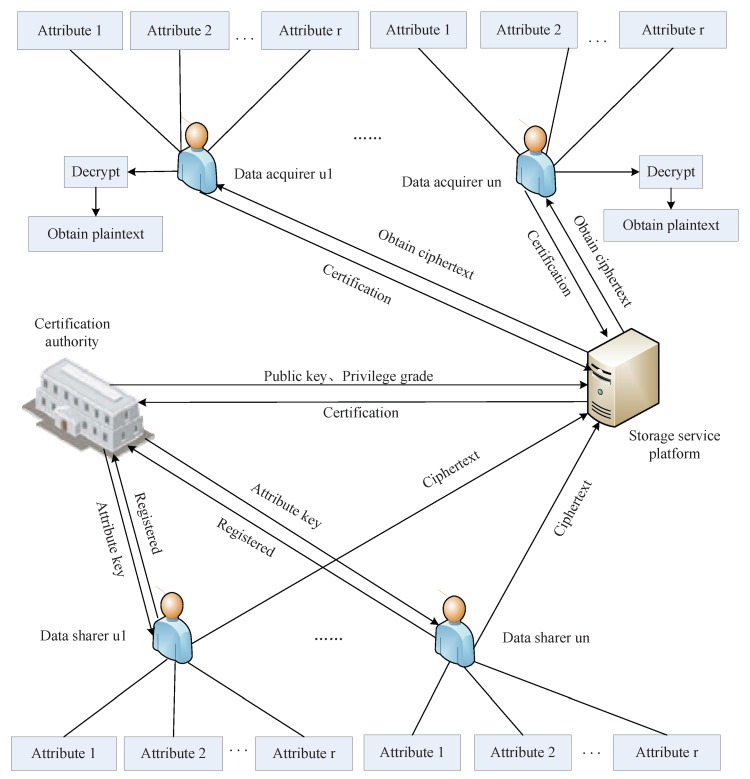
System model diagram.

The system model we designed is shown in Figure 1, which consists of four entities: a certificate authority (CA), many data sharer, many data acquires, and a cloud server (CS). In addition, a user can be either a data acquirer or a data sharer.

The certificate authority (CA) is equivalent to the administrator of the system, who sets system parameters for access control and distributes secret key and privilege level information for the user.

The data sharer uploads his or her own data to the cloud server to share the data with other users. The data content is encrypted before being uploaded to the cloud server.

The data acquirer is an entity that is interested in the data stored in the cloud server, and can view and download related data in the cloud server according to its own access rights.

The cloud server (CS) is a public storage platform that provides data sharers with storage and shared encrypted data. Data requesters can freely access and download data stored in the cloud server according to their own permissions.

### 4.2. Initialization

In this section, we initialize an access control scheme based on ciphertext attribute authentication and threshold policy for Internet of Things. This access control system consists of a certification authority (CA), a cloud server (CS), and network terminal users. CA is a trusted entity used primarily for identity authentication, user registration, and attribute key distribution, and it also generates system public parameters and master keys. CS is an important entity, mainly used for the division of access rights of user encrypted information and the classification and storage of different access rights information. The system model is shown in Figure 1.

In this work, it is supposed that the protocol has *n* network terminals. Let $U=\{u_{1},u_{2},\ldots ,u_{n}\}$ be the set of network terminals. In addition, the corresponding identity set is $ID=\{id_{u_{1}},id_{u_{2}},\ldots ,id_{u_{n}}\}$. CA defines an ordered network attribute set $Attr=\{A_{1},A_{2},\ldots ,A_{j},\ldots ,A_{R}\}$, where $A_{j}<A_{j+1}\left(j<R\right)$ and $R\in N^{*}$ denotes the number of the network attribute. In addition, $attr_{i}=\left\{a_{i,1},a_{i,2},\ldots ,a_{i,r}\right\}$ is the ordered attribute set of network terminal $u_{i}$, where $attr_{i}\subseteq Attr$, $r\in N^{*}$, r≤R and $a_{i,r-1}<a_{i,r}$. *i* denotes the ith terminal and *r* denotes the rth attribute of $u_{i}$.

If the network terminal wants to store encrypted information on the cloud server or access encrypted information on the cloud server, it must register the attributes in the authentication center and obtain corresponding data storage and data access rights.

Assuming $G_{1}$ is an additive group, and the $G_{2}$ is a multiplicative group, they have the same large prime number order *q*, and discrete logarithm over $G_{1}$ and $G_{2}$ are difficult, $g_{1}\in G_{1}$ is a generator of $G_{1}$. Parameter $e:G_{1}\times G_{1}\to G_{2}$ is a computable bilinear mapping. $H_{1}:{\left\{0,1\right\}}^{*}\to Z_{q}^{*},H_{2}:G_{1}\to Z_{q}^{*}$ and $H_{3}:G_{2}\to Z_{q}^{*}$ are three hash functions.

The CA runs the $KeyGen(1^{\lambda })$ algorithm to obtain a public/private key pair $\left(SK_{A},PK_{A}\right)$, where $SK_{A}\in Z_{q}^{*}$ and $PK_{A}=SK_{A}g_{1}$. The any member $u_{i}\in U\left(1\leq i\leq n\right)$ chooses a random positive integer $s_{u_{i}}\in Z_{q}^{*}$ and calculates $sk_{u_{i}}=H_{1}\left(id_{u_{i}}\right)s_{u_{i}}$. $sk_{u_{i}}$ as its private key and the public key $pk_{u_{i}}=g_{1}sk_{u_{i}}$. The system parameters are $params=(PK_{A},q,G_{1},G_{2},g_{1},e,H_{1},H_{2},H_{3})$.

### 4.3. Terminal Users Registration

The terminal users registration of AC-CAATP is depicted in Table 1, and the detailed steps are performed as follows:

(1) CA constructs an *R* degree polynomial(1) by using the elements in the network attribute set $Attr=\{A_{1},A_{2},\ldots ,A_{j},\ldots ,A_{R}\}$ (arranged according to the sequence of attributes specified by the network system) as the coefficients of the polynomial:
  (1) $$f\left(x\right)=\left(x-A_{R}\right)\left(x-A_{R-1}\right)\ldots \left(x-A_{1}\right)=b_{R}x^{R}+b_{R-1}x^{R-1}+\ldots +b_{0}.$$
(2) Each terminal user $u_{i}\left(1\leq i\leq n\right)$ with the attribute set $attr_{i}=\left\{a_{i,1},a_{i,2},\ldots ,a_{i,r}\right\}$ (arranged according to the sequence of attributes specified by the network system) selects a random number $\lambda_{i}\in Z_{q}^{*}\left(\lambda_{i}\neq 1,0\right)$ and calculates $\left\{\left(\lambda_{i}g_{1},a_{i,1}\lambda_{i}g_{1},\ldots ,a_{i,1}^{R}\lambda_{i}g_{1}\right),\left(\lambda_{i}g_{1},a_{i,2}\lambda_{i}g_{1},\ldots ,a_{i,2}^{R}\lambda_{i}g_{1}\right),\ldots ,\left(\lambda_{i}g_{1},a_{i,r}\lambda_{i}g_{1},\ldots ,a_{i,r}^{R}\lambda_{i}g_{1}\right)\right\}$ and $\beta_{i}=\left(a_{i,1}+a_{i,2}+\ldots +a_{i,r}\right)sk_{u_{i}}\lambda_{i}g_{1}$. Then, $u_{i}$ sends $\left\{\left(\lambda_{i}g_{1},a_{i,1}\lambda_{i}g_{1},\ldots ,a_{i,1}^{R}\lambda_{i}g_{1}\right),\left(\lambda_{i}g_{1},a_{i,2}\lambda_{i}g_{1},\ldots ,a_{i,2}^{R}\lambda_{i}g_{1}\right),\ldots ,\left(\lambda_{i}g_{1},a_{i,r}\lambda_{i}g_{1},\ldots ,a_{i,r}^{R}\lambda_{i}g_{1}\right),\beta_{i},pk_{u_{i}}\right\}$ to CA.
(3) After receiving the messages $\left\{\left(\lambda_{i}g_{1},a_{i,1}\lambda_{i}g_{1},\ldots ,a_{i,1}^{R}\lambda_{i}g_{1}\right),\left(\lambda_{i}g_{1},a_{i,2}\lambda_{i}g_{1},\ldots ,a_{i,2}^{R}\lambda_{i}g_{1}\right),\ldots ,\left(\lambda_{i}g_{1},a_{i,r}\lambda_{i}g_{1},\ldots ,a_{i,r}^{R}\lambda_{i}g_{1}\right),\beta_{i},pk_{u_{i}}\right\}$, CA calculates $\gamma_{i}=a_{i,1}\lambda_{i}g_{1}+a_{i,2}\lambda_{i}g_{1}+\ldots +a_{i,r}\lambda_{i}g_{1}$ and verifies the identity of $u_{i}$ by equation $e\left(\beta_{i},g_{1}\right)=e\left(\gamma_{i},pk_{u_{i}}\right)$. If it holds, CA calculates the following formula (2) according to the ploynomial(1):
  (2) $$\begin{array}{c} b_{0}\lambda_{i}g_{1}+b{}_{1}a_{i,1}\lambda_{i}g_{1}+\ldots +b_{R}a_{i,1}^{R}\lambda_{i}g_{1}=f\left(a_{i,1}\right)\lambda_{i}g_{1},\\ b_{0}\lambda_{i}g_{1}+b{}_{1}a_{i,2}\lambda_{i}g_{1}+\ldots +b_{R}a_{i,2}^{R}\lambda_{i}g_{1}=f\left(a_{i,2}\right)\lambda_{i}g_{1},\\ \ldots \\ b_{0}\lambda_{i}g_{1}+b{}_{1}a_{i,r}\lambda_{i}g_{1}+\ldots +b_{R}a_{i,r}^{R}\lambda_{i}g_{1}=f\left(a_{i,r}\right)\lambda_{i}g_{1}. \end{array}$$
  If Equation (2) is equal to 0, this means that $f\left(a_{i,1}\right)=0,f\left(a_{i,2}\right)=0,\ldots ,f\left(a_{i,r}\right)=0$ and $attr_{i}\subseteq Attr$. Then, CA computes $Q_{j}=A_{j}\lambda_{i}g_{1}\left(1\leq j\leq R\right)$ and compares whether the equation $A_{j}\lambda_{i}g_{1}=a_{i,\iota }\lambda_{i}g_{1}\left(1\leq \iota \leq r\right)$ is true. If it holds, CA can determine which attributes the user $u_{i}$ has; according to the corresponding attribute values, CA selects the corresponding attribute parameters $t_{i,1},t_{i,2},\ldots ,t_{i,r}\in Z_{q}^{*}$. It calculates formula (3), and CA divides the permission level according to the number of their attributes and calculates formula (4) as the privilege grade:
  (3) $$\{T_{i,0}=\lambda_{i}g_{1},T_{i,1}=t_{i,1}T_{i,0},T_{i,2}=t_{i,2}T_{i,0},\ldots T_{i,r}=t_{i,r}T_{i,0}\},$$
  (4) $$\eta_{i,h}=SK_{A}\left(t_{i,1}+t_{i,2}+\ldots +t_{i,r}\right)g_{1}.$$
  Then, CA sends $\left\{\eta_{i,h},T_{i,1},T_{i,2},\ldots ,T_{i,r}\right\}$ to the register terminal $u_{i}$ and secretly saves parameter $\gamma_{i}$. (Note that, for any two attributes $a_{i,k}$ and $a_{j,l}$ of different members of $u_{i}$ and $u_{j}\left(i\neq j\right)$, if $a_{i,k}=a_{j,l}$, then $t_{i,k}=t_{j,l}$).
(4) After receiving the messages $\left\{\eta_{i,h},T_{i,1},T_{i,2},\ldots ,T_{i,r}\right\}$ from CA, $u_{i}\left(1\leq i\leq n\right)$ calculates formula (5) and verifies the identity of CA by equation $e\left(\eta_{i,h},g_{1}\right)=e\left(\varepsilon_{i},PK_{A}\right)$. If it holds, $u_{i}$ computes the following formula (6) according to formula (3) and obtains the attribute permission values $\left\{K_{i,1},K_{i,2},\ldots ,K_{i,r}\right\}$ and the privilege level $\eta_{i,h}$:
  (5) $$\varepsilon_{i}=\lambda_{i}^{-1}T_{i}+\lambda_{i}^{-1}T_{i,2}+\ldots +\lambda_{i}^{-1}T_{i,r}=\left(t_{i,1}+t_{i,2}+\ldots +t_{i,r}\right)g_{1},$$
  (6) $$K_{i,1}=\lambda_{i}^{-1}T_{i,1}=t_{i,1}g_{1},K_{i,2}=\lambda_{i}^{-1}T_{i,2}=t_{i,2}g_{1},\ldots ,K_{i,r}=\lambda_{i}^{-1}T_{i,r}=t_{i,r}g_{1}.$$
  $u_{i}$ sends messages $\left\{u_{i},pk_{u_{i}},\eta_{i,h}\right\}$ to CA indicating that it has successfully registered.
(5) After receiving the messages $\left\{u_{i},pk_{u_{i}},\eta_{i,h}\right\}$ from $u_{i}$, CA verifies the messages and sends it to CS.

With the above steps, all the terminals $u_{i}\left(1\leq i\leq n\right)$ register successfully. In addition, CA can obtain the attribute information from all the registration terminals $u_{i}\left(1\leq i\leq n\right)$. CA divides the permission levels of group members according to the number of attributes. Then, CA can build a terminal users registration information table (as shown in Table 2) and share the information resource with CS, which is used for querying user rights and access control of resource permissions.

### 4.4. Resource Encryption Storage

Each terminal user can encrypt their shared resources and upload them to the cloud server. Any member $u_{j}\left(1\leq j\leq n\right)$ with the attribute set $attr_{j}=\left\{a_{j,1},a_{j,2},\ldots ,a_{j,r}\right\}$ and the privilege value $\eta_{j,h}=SK_{A}\left(t_{j,1}+t_{j,2}+\ldots +t_{j,r}\right)g_{1}$ in the network wants to share resources to the members who have the same or higher privileges than him. He can do the following steps to encrypt resources and upload them to the cloud server:

(1) $u_{j}$ gets the information $T_{j,1},\ldots ,T_{j,r}$ from the information in Table 1 and computes formula (7) and formula (8):
  (7) $$T_{pub,j}=T_{j,0}=\lambda_{j}g_{1},$$
  (8) $$T_{pri}=\sum_{\tau =1}^{r}T_{j,\tau }=\sum_{\tau =1}^{r}t_{j,\tau }\lambda_{j}g_{1}=\left(t_{j,1}+\ldots +t_{j,r}\right)\lambda_{j}g_{1}.$$
(2) $u_{j}$ selects $m_{j}\in Z_{p}^{*}$ randomly, then calculate formulas (9), (10), and (11), according to formulas (6), (7), and (8) and constructs a (r−1)−th degree polynomial(12) according to the attribute permission values $\left\{K_{j,1},K_{j,2},\ldots ,K_{j,r}\right\}$ that it kept before and $f\left(0\right)=M_{j}$; then, it computes formula (13) according to formula (11) and $\varphi_{j}=sk_{u_{j}}\left(y_{j,1}+y_{j,2}+\ldots +y_{j,r}\right)$. $u_{j}$ uses $PK_{g-u_{j}}=\left(p_{u_{j}},\eta_{j,h}\right)$ as encryption key and $SK_{g-u_{j}}=M_{j}$ as decryption key:
  (9) $$p_{u_{j}}=m_{j}T_{j,0}=m_{j}\lambda_{j}g_{1},$$
  (10) $$M_{j}=m_{j}T_{pri},$$
  (11) $$w_{j,1}=H_{2}\left(K_{j,1}\right),w_{j,2}=H_{2}\left(K_{j,2}\right),\ldots ,w_{j,r}=H_{2}\left(K_{j,r}\right),$$
  (12) $$f\left(x\right)=m_{j}K_{j,r-1}x^{r-1}+\ldots +m_{j}K_{j,1}x+M_{j},$$
  (13) $$f\left(w_{j,1}\right)=y_{j,1},f\left(w_{j,2}\right)=y_{j,2},\ldots ,f\left(w_{j,r}\right)=y_{j,r}.$$
(3) $u_{j}$ encrypts its shared resources information $m\in M^{*}$ ($M^{*}$: plaintext space) with encryption key $PK_{g-u_{j}}=\left(p_{u_{j}},\eta_{j,h}\right)$, which is that $u_{j}$ chooses a random number $\varsigma_{j}\in Z_{p}^{*}$, and calculates formulas (14), (15), and (16) according to formulas (4) and (9), the corresponding ciphertext information is $c_{j}=\left(\upsilon_{j},V_{j}\right)$:
  (14) $$H_{3}{\left(e\left(p_{u_{j}},\eta_{j,h}\right)\right)}^{\varsigma_{j}},$$
  (15) $$\upsilon_{j}=\varsigma_{j}PK_{A},$$
  (16) $$V_{j}=m⊕H_{3}(\left(e{\left(p_{u_{j}},\eta_{j,h}\right)}^{\varsigma_{j}}\right).$$
  Then, $u_{j}$ uploads the shared ciphertext information $c_{j}=\left(\upsilon_{j},V_{j}\right)$. The plaintext information of the keywords of the shared resource and the related description of the resource (search for related resources primarily for resource visitors), encryption key $PK_{g-u_{j}}=\left(p_{u_{j}},\eta_{j,h}\right)$ and related calculation parameters $\left\{\left(y_{j,1},y_{j,2},\ldots ,y_{j,r}\right),\left(T_{j,1},T_{j,2},\ldots ,T_{j,r}\right),\varphi_{j},pk_{u_{i}},\eta_{j,h}\right\}$ to the CS. CS verifies the identity of $u_{j}$ by the equation $e\left(\left(y_{j,1}+y_{j,2}+\ldots +y_{j,r}\right),pk_{u_{i}}\right)=e\left(\varphi_{j},g_{1}\right)$. If it holds, CS publishes the information $\left\{u_{j},pk_{u_{j}},Keywords_{j},D_{j},PK_{g-u_{j}},c_{j},\eta_{i,h},\left(y_{j,1},y_{j,2},\ldots ,y_{j,r}\right)\right\}$ on the public display platform as shown in Table 2, where $Keywords_{j}$ is the keywords of the shared resource, and $D_{j}$ is the related description of the resource.

### 4.5. Resource Access and Sharing

(1) Each user $u_{i}(1\leq i\leq n,i\neq j)$ in the cloud system wants to access resources in the system; it can search for the corresponding ciphertext resource according to the keyword and related content description and can view the provider of the resource and access rights that should be available to access the resource.
(2) If $u_{i}$ wants to access certain resources and has the access rights of the resource, $u_{i}$ computes formula (17) according to formula (5), and sends the messages $\left(pk_{u_{i}},\eta_{i,h},\sigma_{i}\right)$ to CS:
  (17) $$\sigma_{i}=sk_{u_{i}}\varepsilon_{i}.$$
  Then, CS verifies the identity of $u_{i}$ by the equation $e\left(\eta_{i,h},pk_{u_{i}}\right)=e\left(\sigma_{i},PK_{A}\right)$. If it holds, CS opens the corresponding resource link.
(3) $u_{i}$ downloads the corresponding ciphertext resource $c_{j}=\left(\upsilon_{j},V_{j}\right)$ from the CS. It can compute the corresponding attribute permission values $\left\{K_{j,1},K_{j,2},\ldots ,K_{j,r}\right\}$ according to the right parameters $\left(T_{j,1},T_{j,2}\ldots ,T_{j,r}\right)$ and corresponding threshold value $\left(y_{j,1},y_{j,2},\ldots ,y_{j,r}\right)$. It computes $w_{i,1}=H_{2}\left(K_{i,1}\right),w_{i,2}=H_{2}\left(K_{i,2}\right),\ldots ,w_{i,r}=H_{2}\left(K_{i,r}\right)$. $u_{i}$ constructs polynomial (18) according to the information $\left\{\left(w_{i,1},y_{j,1}\right),\left(w_{i,2},y_{j,2}\right),\ldots ,\left(w_{i,r},y_{j,r}\right)\right\}$ and Lagrange theorem:
  (18) $$f\left(x\right)=\sum_{\chi =1}^{r}\left(\prod_{1\leq \varpi \leq r,\varpi \neq \chi }\frac{x-w_{i,\varpi }}{w_{i,\chi }-w_{i,\varpi }}\right)y_{j,\chi }.$$
  In addition, it computes the constant term $M_{i}=f\left(0\right)=\sum_{\chi =1}^{r}\left(\prod_{1\leq \varpi \leq r,\varpi \neq \chi }\frac{-w_{i,\varpi }}{w_{i,\chi }-w_{i,\varpi }}\right)y_{j,\chi }=M_{j}$ as its decryption key. $u_{i}$ can also obtain the encryption key $PK_{g-u_{k}}=\left(p_{u_{k}},\eta_{k,h}\right)=\left(p_{u_{j}},\eta_{j,h}\right)$ from Table 2.
(4) Anyone $u_{i}\left(1\leq i\leq n,i\neq j\right)$ in the network system can calculate $m=V_{j}⊕H_{3}\left(e\left(\upsilon_{j},M_{i}\right)\right)$ from ciphertext $c_{j}=\left(\upsilon_{j},V_{j}\right)$, with a valid decryption key $M_{i}$.

## 5. Correctness and Security Analysis

This section mainly discusses some of the performances of AC-CAATP protocol. First, it proves the correctness of AC-CAATP protocol, then discusses the security of AC-CAATP protocol, and finally analyzes the performance of AC-CAATP protocol.

### 5.1. Correctness

The proof of the correctness of AC-CAATP protocol is in the following theorems.

**Theorem** **1.** *Any legal user $u_{i}\left(1\leq i\leq n\right)$ in the system can download the ciphertext resource corresponding to the access rights. This means if $\eta_{i,h}>\eta_{j,h}$, $u_{i}\left(1\leq i\leq n\right)$ can download the ciphertext resource $c_{j}$.*

**Proof.**  We assume that $u_{i}$ has a set of attributes $attr_{i}=\left\{a_{i,1},a_{i,2},\ldots ,a_{i,r}\right\}$ and the correspond attribute weight parameter is $\varepsilon_{i}=\left(t_{i,1}+t_{i,2}+\ldots +t_{i,r}\right)g_{1}$. $u_{i}$ declares that it has access hierarchical $\eta_{i,h}$. It signs the parameter $\varepsilon_{i}$ and the signed message is $\sigma_{i}=sk_{u_{i}}\varepsilon_{i}$. Then, it sends the messages $\left(pk_{u_{i}},\eta_{i,h},\sigma_{i}\right)$ to CS. According to the protocol AC-CAATP, CS verifies the identity of $u_{i}$ by the equation $e\left(\eta_{i,h},pk_{u_{i}}\right)=e\left(\sigma_{i},PK_{A}\right)$. If it holds, CS opens the corresponding resource link. Since $\eta_{i,h}=SK_{A}\left(t_{i,1}+t_{i,2}+\ldots +t_{i,r}\right)g_{1}$ and $\sigma_{i}=sk_{u_{i}}\varepsilon_{i}$, according to the characteristics of bilinear mapping, there are

$$\begin{array}{c} e(\eta_{i,h},pk_{u_{i}}),\\ \;=e(SK_{A}\left(t_{i,1}+t_{i,2}+\ldots +t_{i,r}\right)g_{1},sk_{u_{i}}g_{1})\\ \;=e{\left(\left(t_{i,1}+t_{i,2}+\ldots +t_{i,r}\right)g_{1},g_{1}\right)}^{SK_{A}sk_{u_{i}}}\\ \;=e(sk_{u_{i}}\left(t_{i,1}+t_{i,2}+\ldots +t_{i,r}\right)g_{1},SK_{A}g_{1})\\ \;=e(\sigma_{i},PK_{A}). \end{array}$$

The attribute weight parameter $\varepsilon_{i}=\left(t_{i,1}+t_{i,2}+\ldots +t_{i,r}\right)g_{1}$ is signed by $u_{i}$ and CS. This means that CS can ensure $u_{i}$ has the access hierarchical $\eta_{i,h}$. Then, CS opens the corresponding resource link $c_{j}$ for $u_{i}$. $u_{i}$ can download the ciphertext resource $c_{j}$.  □

**Theorem** **2.** Any member $u_{i}\left(1\leq i\leq n\right)$ with access right $\eta_{i,h}$, if $\eta_{i,h}\geq \eta_{j,h}$, $u_{i}$ can access the corresponding resources $m\in M^{*}$ belonging to $u_{j}\left(1\leq j\leq n,i\neq j\right)$. This means that member $u_{i}\left(1\leq i\leq n\right)$ can decrypt the ciphertext information $c_{j}$ that was encrypted by member $u_{j}\left(1\leq j\leq n,i\neq j\right)$ using the encryption key $PK_{g-u_{j}}=\left(p_{u_{j}},\eta_{j,h}\right)$.

**Proof.**  If $u_{i}$ has the access right $\eta_{i,h}$ and $\eta_{i,h}\geq \eta_{j,h}$, then $u_{i}$ has the attribute permission values $K_{i,1}=t_{i,1}g_{1}=K_{j,1},K_{i,2}=t_{i,2}g_{1}=K_{j,2},\ldots ,K_{i,r}=t_{i,r}g_{1}=K_{j,r}$, It can compute $w_{i,1}=H_{2}\left(K_{i,1}\right),w_{i,2}=H_{2}\left(K_{i,2}\right),\ldots ,w_{i,r}=H_{2}\left(K_{i,r}\right)$. $u_{i}$ can construct a polynomial $f\left(x\right)=\sum_{\chi =1}^{r}\left(\prod_{1\leq \varpi \leq r,\varpi \neq \chi }\frac{x-w_{i,\varpi }}{w_{i,\chi }-w_{i,\varpi }}\right)y_{j,\chi }$ according to the information $\left\{\left(w_{i,1},y_{j,1}\right),\left(w_{i,2},y_{j,2}\right),\ldots ,\left(w_{i,r},y_{j,r}\right)\right\}$ from registration information table and the Lagrange theorem, and compute the constant term $M_{i}=f\left(0\right)=\sum_{\chi =1}^{r}\left(\prod_{1\leq \varpi \leq r,\varpi \neq \chi }\frac{-w_{i,\varpi }}{w_{i,\chi }-w_{i,\varpi }}\right)y_{j,\chi }=M_{j}$ as its the decryption key.

Since $T_{pub,j}=T_{j,0}=\lambda_{j}g_{1}$, $T_{pri}=\left(t_{j,1}+\ldots +t_{j,r}\right)\lambda_{j}g_{1}$, $\eta_{j,h}=SK_{A}\left(t_{j,1}+t_{j,2}+\ldots +t_{j,r}\right)g_{1}$, $p_{u_{j}}=m_{j}\lambda_{j}g_{1}$, $M_{j}=m_{j}T_{pri}$ and the ciphertext information is $c_{j}=\left(\upsilon_{j},V_{j}\right)$, where $\upsilon_{j}=\varsigma_{j}PK_{A}$ and $V_{j}=m⊕H_{3}(\left(e{\left(p_{u_{j}},\eta_{j,h}\right)}^{\varsigma_{j}}\right)$. Then, $u_{i}$ uses its own solved key $M_{i}=M_{i}=m_{j}T_{pri}$ to do the following calculation:

$$\begin{array}{c} V_{j}⊕H_{3}\left(e\left(\upsilon_{j},M_{i}\right)\right)\\ \;=m⊕H_{3}\left(e{\left(p_{u_{j}},\eta_{j,h}\right)}^{\varsigma_{j}}\right)⊕H_{3}\left(e\left(\upsilon_{j},M_{i}\right)\right)\\ =m⊕H_{3}\left(e{\left(m_{j}\lambda_{j}g_{1},SK_{A}\left(t_{j,1}+t_{j,2}+\ldots +t_{j,r}\right)g_{1}\right)}^{\varsigma_{j}}\right)⊕H_{3}\left(e\left(\varsigma_{j}PK_{A},m_{j}T_{pri}\right)\right)\\ =m⊕H_{3}\left(e{\left(m_{j}g_{1},\left(t_{j,1}+t_{j,2}+\ldots +t_{j,r}\right)PK_{A}\right)}^{\lambda_{j}\varsigma_{j}}\right)⊕H_{3}\left(e\left(\varsigma_{j}PK_{A},m_{j}T_{pri}\right)\right)\\ =m⊕H_{3}\left(e{\left(m_{j}g_{1},\left(t_{j,1}+t_{j,2}+\ldots +t_{j,r}\right)PK_{A}\right)}^{\lambda_{j}\varsigma_{j}}\right)⊕H_{3}\left(e{\left(PK_{A},m_{j}T_{pri}\right)}^{\varsigma_{j}}\right)\\ =m⊕H_{3}\left(e{\left(m_{j}g_{1},\left(t_{j,1}+t_{j,2}+\ldots +t_{j,r}\right)PK_{A}\right)}^{\lambda_{j}\varsigma_{j}}\right)⊕H_{3}\left(e{\left(PK_{A},m_{j}\left(t_{j,1}+\ldots +t_{j,r}\right)\lambda_{j}g_{1}\right)}^{\varsigma_{j}}\right)\\ =m⊕H_{3}\left(e{\left(m_{j}g_{1},PK_{A}\right)}^{\left(t_{j,1}+t_{j,2}+\ldots +t_{j,r}\right)\lambda_{j}\varsigma_{j}}\right)⊕H_{3}\left(e{\left(PK_{A},m_{j}g_{1}\right)}^{\left(t_{j,1}+\ldots +t_{j,r}\right)\lambda_{j}\varsigma_{j}}\right)\\ =m. \end{array}$$

Thus, $u_{i}$ can decrypt the ciphertext information $c_{j}$ and get the corresponding plaintext resources *m*.  □

### 5.2. Security Analysis

**Theorem** **3.** *Users with low access rights cannot access resources with higher permission grades than themselves. This means $u_{i}\left(1\leq i\leq n\right)$ with access right $\eta_{i,h}$, if $\eta_{i,h}<\eta_{j,h}$, $u_{i}$ cannot get decryption key $M_{j}$ by solving polynomial functions to decrypt the ciphertext information $c_{j}$ and get the corresponding plaintext resources m.*

**Proof.**  If $\eta_{i,h}<\eta_{j,h}$, this means that $u_{i}$ does not have enough attribute permission values $K_{j,1}=t_{j,1}g_{1},K_{j,2}=t_{j,2}g_{1},\ldots ,K_{j,r}=t_{j,r}g_{1}$. It cannot compute $w_{j,1}=H_{2}\left(K_{j,1}\right),w_{j,2}=H_{2}\left(K_{j,2}\right),\ldots ,w_{j,r}=H_{2}\left(K_{j,r}\right)$ to get the point pair $\left\{\left(w_{j,1},y_{j,1}\right),\left(w_{j,2},y_{j,2}\right),\ldots ,\left(w_{j,r},y_{j,r}\right)\right\}$ and construct a polynomial $f\left(x\right)=\sum_{\chi =1}^{r}\left(\prod_{1\leq \varpi \leq r,\varpi \neq \chi }\frac{x-w_{j,\varpi }}{w_{j,\chi }-w_{j,\varpi }}\right)y_{j,\chi }$.  □

**Lemma** **1.** *If $w_{j,1},w_{j,2},\ldots ,w_{j,r}$ are different numbers in the number field F, $y_{j,1},y_{j,2},\ldots ,y_{j,r}$ are any set of numbers in the number field F. There is a unique polynomial $f\left(x\right)=\sum_{\chi =1}^{r}\left(\prod_{1\leq \varpi \leq r,\varpi \neq \chi }\frac{x-w_{j,\varpi }}{w_{j,\chi }-w_{j,\varpi }}\right)y_{j,\chi }$ in which the degree is no greater than r−1, such that $f\left(w_{j,\tau }\right)=y_{j,\tau }$, where τ=1,2,…,r.*

**Proof.**  Assume there are two polynomials f(x) and g(x) in F(x), their degrees no greater than r−1, and they all satisfy the equations: $f\left(w_{j,\tau }\right)=y_{j,\tau }$, $g\left(w_{j,\tau }\right)=y_{j,\tau }$, where τ=1,2,…,r.  □

Let ∂(x)=f(x)−g(x), if f(x)≠g(x), then ∂(x)≠0. ∂(x) is a polynomial with a degree no greater than r−1 and the polynomial has *r* solutions, which is impossible. Thus, ∂(x)=0, which means that f(x)=g(x), and polynomial f(x) is unique.

**Lemma** **2.** *Polynomial f(x) has a unique solution.*

**Proof.**  Assume $f\left(x\right)=c_{r-1}x^{r-1}+c_{r-2}x^{r-2}+\ldots +c_{0}$, for $f\left(w_{j,\tau }\right)=y_{j,\tau }\left(1\leq \tau \leq r\right)$, there are

$$\left\{\begin{array}{c} c_{0}+c_{1}w_{j,1}^{1}+c_{2}w_{j,1}^{2}+\ldots +c_{r-1}w_{j,1}^{r-1}=y_{j,1}\\ c_{0}+c_{1}w_{j,2}^{1}+c_{2}w_{j,2}^{2}+\ldots +c_{r-1}w_{j,2}^{r-1}=y_{j,2}\\ \ldots \ldots \ldots \ldots \ldots \ldots \ldots \ldots \ldots \ldots \ldots \ldots \ldots \ldots \ldots \ldots \ldots \ldots \ldots .\\ c_{0}+c_{1}w_{j,r}^{1}+c_{2}w_{j,r}^{2}+\ldots +c_{r-1}w_{j,r}^{r-1}=y_{j,r}. \end{array}\right.$$

This is a linear system of equations with unknown numbers $c_{0},c_{1}\ldots c_{r}$. Its coefficient determinant is as follows:

$$\left|A\right|=\left|\begin{array}{ccccc} 1 & w_{j,1}^{1} & w_{j,1}^{2} & \ldots & w_{j,1}^{r-1}\\ 1 & w_{j,2}^{1} & w_{j,2}^{2} & \ldots & w_{j,2}^{r-1}\\ \ldots & \ldots & \ldots & \ldots & \ldots \\ 1 & w_{j,r}^{1} & w_{j,r}^{2} & \ldots & w_{j,r}^{r-1} \end{array}\right|=\left|\begin{array}{cccc} 1 & 1 & \ldots & 1\\ w_{j,1}^{1} & w_{j,2}^{1} & \ldots & w_{j,r}^{1}\\ w_{j,1}^{2} & w_{j,2}^{2} & \ldots & w_{j,r}^{2}\\ \ldots & \ldots & \ldots & \ldots \\ w_{j,1}^{r-1} & w_{j,2}^{r-1} & \ldots & w_{j,r}^{r-1} \end{array}\right|=\prod_{r\geq i>\tau \geq 1}\left(w_{j,i}-w_{j,\tau }\right).$$

This a Vandermonde determinant, for $w_{j,i}\neq w_{j,\tau }$, so $\left|A\right|\neq 0$, and, therefore, the linear equations have a unique solution $c_{0},c_{1}\ldots c_{r}$.

If $\eta_{i,h}<\eta_{j,h}$ this means that $u_{i}$ does not have enough attribute permission values $K_{j,1}=t_{j,1}g_{1},K_{j,2}=t_{j,2}g_{1},\ldots ,K_{j,r}=t_{j,r}g_{1}$. Assume $u_{i}$ does not have attribute permission value $K_{j,r}=t_{j,r}g_{1}$, that is, it cannot compute $w_{j,r}$. According to the point pair $\left\{\left(w_{j,1},y_{j,1}\right),\left(w_{j,2},y_{j,2}\right),\ldots ,\left(w_{j,r-1},y_{j,r-1}\right)\right\}$, $u_{i}$ cannot construct polynomial $c_{0}+c_{1}w_{j,r}^{1}+c_{2}w_{j,r}^{2}+\ldots +c_{r-1}w_{j,r}^{r-1}=y_{j,r}$, and only construct a linear system of equations with unknown numbers $c_{0},c_{1}\ldots c_{r}$:

$$\left\{\begin{array}{c} c_{0}+c_{1}w_{j,1}^{1}+c_{2}w_{j,1}^{2}+\ldots +c_{r-1}w_{j,1}^{r-1}=y_{j,1}\\ c_{0}+c_{1}w_{j,2}^{1}+c_{2}w_{j,2}^{2}+\ldots +c_{r-1}w_{j,2}^{r-1}=y_{j,2}\\ \ldots \ldots \ldots \ldots \ldots \ldots \ldots \ldots \ldots \ldots \ldots \ldots \ldots \ldots \ldots \ldots \ldots \ldots \ldots \\ c_{0}+c_{1}w_{j,r-1}^{1}+c_{2}w_{j,r-1}^{2}+\ldots +c_{r-1}w_{j,r-1}^{r-1}=y_{j,r-1}. \end{array}\right.$$

There is no solution to this linear system of equations. Thus, $u_{i}$ cannot get decryption key $M_{j}$ by solving polynomial functions to decrypt the ciphertext information $c_{j}$.  □

**Theorem** **4.** proposed AC-CAATP protocol is security against passive adversary under the DBDH problem assumption. That is, under the DBDH assumption, for any $a,b,c\in Z_{q}^{*}$, $g_{1}\in G_{1}$, $g_{2}\in G_{2}$ and $\pi \in G_{2}$, there are two computationally indistinguishable tuples $\left(g_{1},g_{2},ag_{1},bg_{1},cg_{1},e{\left(g_{1},g_{1}\right)}^{abc}\right)$ and $\left(g_{1},g_{2},ag_{1},bg_{1},cg_{1},\pi \right)$. Even if adversary C obtains relevant information $\left\{\eta_{j,h},T_{j,1},T_{j,2},\ldots ,T_{j,r}\right\}$ from the registration process of AC-CAATP in Table 1 and the information $\left\{u_{j},pk_{u_{j}},Keywords_{j},D_{j},PK_{g-u_{j}},c_{j},\eta_{i,h},\left(y_{j,1},y_{j,2},\ldots ,y_{j,r}\right)\right\}$ through the public display platform in Table 2, it cannot obtain the plaintext information $m=V_{j}⊕H_{3}\left(e\left(\upsilon_{j},M_{j}\right)\right)$ without the decryption key $M_{j}$.

**Proof.**  Since $T_{j,\tau }=t_{j,\tau }\lambda_{j}g_{1}\left(0\leq \tau \leq r\right)$, $T_{pri}=\left(t_{j,1}+\ldots +t_{j,r}\right)\lambda_{j}g_{1}$, $\eta_{j,h}=SK_{A}\left(t_{j,1}+t_{j,2}+\ldots +t_{j,r}\right)g_{1}$, $p_{u_{j}}=m_{j}\lambda_{j}g_{1}$, $M_{j}=m_{j}T_{pri}$, where $m_{j}\in Z_{p}^{*}$. The ciphertext information is $c_{j}=\left(\upsilon_{j},V_{j}\right)$, where $\upsilon_{j}=\varsigma_{j}PK_{A}$, $\varsigma_{j}\in Z_{p}^{*}$ and $V_{j}=m⊕H_{3}(\left(e{\left(p_{u_{j}},\eta_{j,h}\right)}^{\varsigma_{j}}\right)$. C does the following algorithm A; it selects $\left(\rho_{1},\rho_{2},\rho_{3}\right)$ from $Z_{p}^{*}$ randomly and computes $f_{1}=\rho_{1}T_{j,1}=\rho_{1}\lambda_{j}t_{j,1}g_{1},f_{2}=\rho_{1}T_{j,2}=\rho_{1}\lambda_{j}t_{j,2}g_{1},f_{r}=\rho_{1}T_{j,r}=\rho_{1}\lambda_{j}t_{j,r}g_{1}$, ${M_{j}}^{\prime }=f_{1}+f_{2}+\ldots +f_{r}=\rho_{1}\left(t_{j,1}+t_{j,1}+\ldots +t_{j,1}\right)\lambda_{j}g_{1}=\rho_{1}ag_{1}$, $v_{j}^{\prime }=\rho_{2}PK_{A}$, $\eta_{j,h}^{\prime }=\rho_{3}PK_{A}$ and $p_{u_{j}}^{\prime }=\rho_{1}T_{j,0}=\rho_{1}cg_{1}$. If $e{\left(p_{u_{j}},\eta_{j,h}\right)}^{\varsigma_{j}}=e\left(M_{j}^{\prime },V_{j}^{\prime }\right)$, this means $\rho_{1}=m_{j}$, $\rho_{2}=\varsigma_{j}$ and $\rho_{2}=t_{j,1}+t_{j,1}+\ldots +t_{j,1}$. Then, we can construct another algorithm $A^{\prime }$ to call A to efficiently distinguish $\left(g_{1},g_{2},ag_{1},bg_{1},cg_{1},e{\left(g_{1},g_{1}\right)}^{abc}\right)$ and $\left(g_{1},g_{2},ag_{1},bg_{1},cg_{1},\pi \right)$, where $ag_{1}=\left(T_{j,1}+T_{j,2}+\ldots +T_{j,r}\right)=\left(t_{j,1}+t_{j,2}+\ldots +t_{j,r}\right)\lambda_{j}g_{1}$, $bg_{1}=V_{j}=\varsigma_{j}PK_{A}=\rho_{3}PK_{A}$ and $cg_{1}=T_{j,0}=\lambda_{j}g_{1}$, which is a contradiction for the DBDH problem assumption. Thus, the proposed protocol is secure against passive attacks under the DBDH problem assumption.  □

**Theorem** **5.** *Our proposed scheme can defend against collusion attacks. Anyone who does not have sufficient attribute rights can’t collude to access resources beyond their access rights.*

**Proof.**  On the one hand, each member’s permission parameters are different, even if they are the same attribute; this means, even if $T_{j,1}=T_{j,1}$, $a_{i,1}$ is not equal to $a_{j,1}$, and $u_{i}$ cannot obtain the attribute $a_{j,1}$ from $T_{j,1}$ in CS platform. On the other hand, each member obtains a privilege grade $\eta_{i,h}$ according to its own attribute when registering the identity. When accessing the resource, the CS perform joint authentication on the identity and privilege grade of the resource visitor by the equation $e\left(\eta_{i,h},pk_{u_{i}}\right)=e\left(\sigma_{i},PK_{A}\right)$, and prohibits the user to access certain resources for which its privilege grade does not meet the requirements for accessing the resource.  □

## 6. Efficiency Analysis

Computational consumption, storage space, and communication load are three important indicators for measuring the performance of access control protocols. According to the analytical data provided by [27], this section compares the proposed scheme with [27,31] in the three performance indicators.

References [27,31] adopt a tree-structured access control scheme. For discussion conveniences, according to the expression of [31], suppose $n_{u}$ is the average number of attributes per user, $n_{c}$ is the average number of attributes associated with the policy tree of ciphertext, |tr| is the average number of translation nodes in a ciphertext, and $\left|tr_{Attr}\right|$ is the average number of necessary translation nodes for the network attribute set Attr.

### 6.1. Computation Overhead

In terms of computing load, the two most important calculations include bilinear pairing operation and exponential operation, since, compared with pairing and exponentiation operation, the cost of addition and multiplication operation can be neglected. Assume there are *n* members participating in system resource sharing. $T_{bp}$ denotes the cost of the bilinear pairing operation on group $G_{1}$, $T_{exp}$ denotes the cost of the exponentiation operation, and $n_{c,u}$ represents the average number of attributes that each user uses to decrypt the ciphertext. $n_{c,Attr}$ represents the average number of attributes used to decrypt ciphertext by a set of users with attribute set Attr, and |Attr| denotes the number of attributes in set Attr. |nl| represents the average number of non-leaf nodes when computing the secret from leaf nodes to root node according to access policies. The computation load comparison for our scheme and other two schemes as shown in Table 3.

From Table 3, for the comparative analysis of the five schemes in terms of the total calculation amount, the total calculation amount of our scheme is the smallest, followed by the scheme Zhong et al. [19]. The calculation amounts of Xue et al. [31] and Li et al. [15] are relatively large. In the initialization phase, because the terminal nodes of the IoT network may be increased, the calculation amount of Xue et al. [31] increases linearly with the increase of network nodes. Our scheme is larger than that of the other three schemes, mainly because our scheme performs attribute encryption authentication in the initialization phase, ensuring that personal attributes are not leaked, protecting personal privacy and higher security. The other four schemes do not have this feature. In the KeyGen phase, our scheme is the smallest, followed by the scheme Bethencourt et al. and Xue et al. [31]. They have the same calculation amount. The scheme of Li et al. [15] is the largest. In the Encrypt and Decrypt phase, our scheme and Zhong et al. [19] are the smallest, followed by the scheme of Bethencourt et al. [27] and Li et al. [15]. The calculation amount of Xue et al. [31] is the largest.

### 6.2. Computation Time Cost

**Figure 2:**
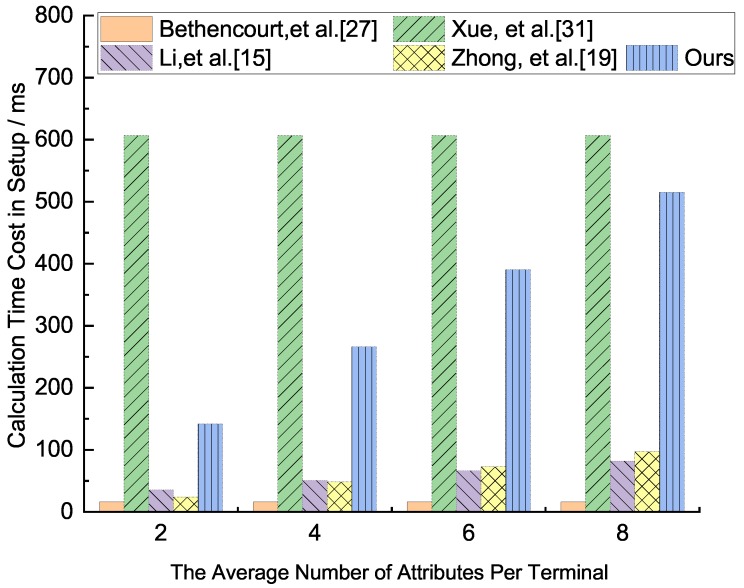
Calculation time cost comparison analysis in the setup phase of the five protocols.

**Figure 3:**
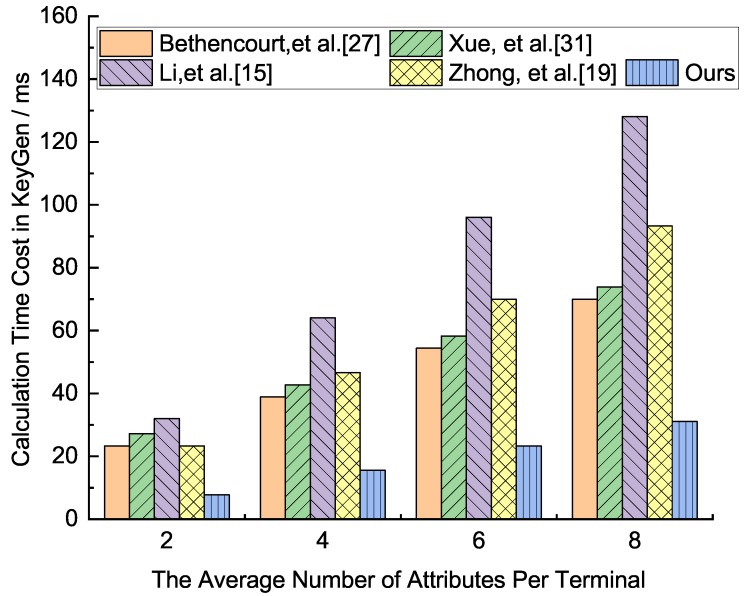
Calculation time cost comparison analysis in the key generation phase of the five protocols.

**Figure 4:**
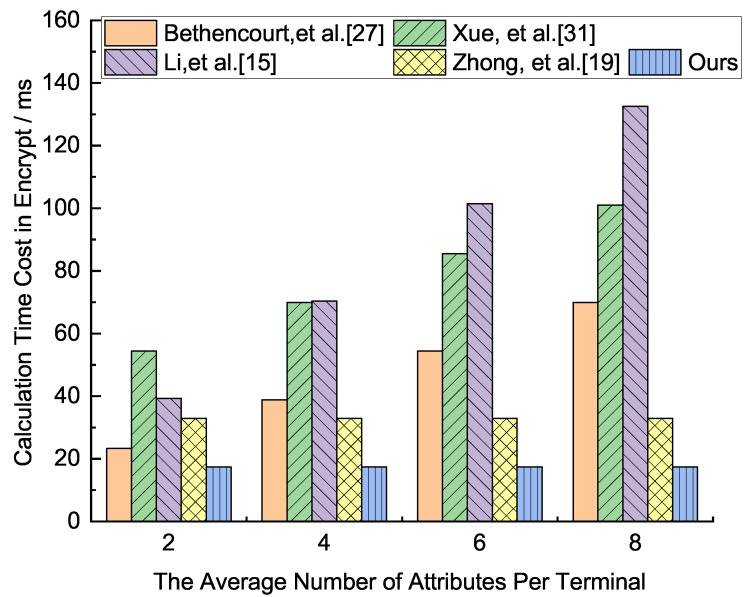
Calculation time cost comparison analysis in the encryption phase of the five protocols.

**Figure 5:**
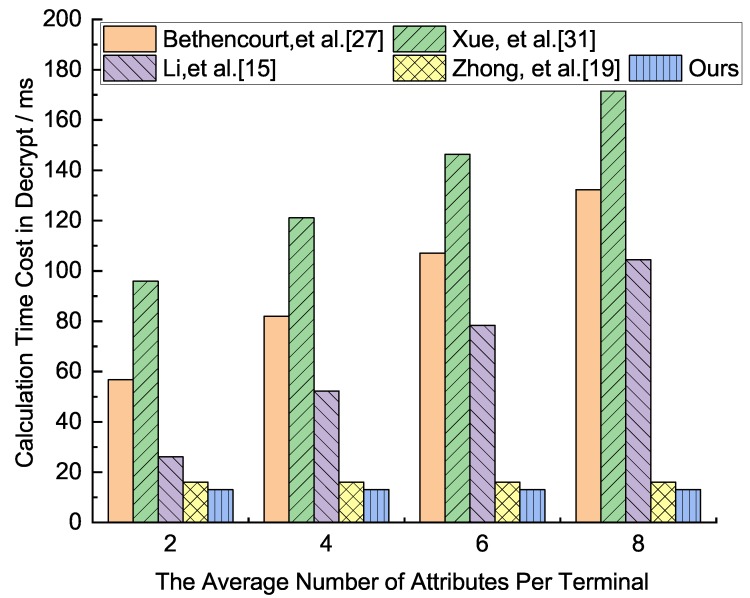
Calculation time cost comparison analysis in the decryption phase of the five protocols.

We analyze how the execution time as the number of node attributes grows in each phase, which runs the related algorithm through program pbc-0.5.12 provided by the Pairing Based Cryptography Library(PBC), on an environment of Intel(R) Core(TM)2 Duo E8400 CPU(3.00 GHz) (LENOVO (Being) LIMITED, Beijing, China ), Ubuntu 10.04, the average run time of the multiplication on $G_{1}$ is 0.016 ms, and the average exponent operation time of $G_{1}$ and $G_{2}$ are 3.886 ms and 0.489 ms, respectively, and the average run time of the bilinear pair is 4.354 ms. Because the execution time of multiplication is about 0.005 times that of the other three algorithms, the multiplication operation can be ignored in our analysis. For the convenience of discussion, we assume that the number of nodes in the network is 150, and the number of attributes in the network attribute set Attr is 8, all the attributes constitute a binary policy tree, all the attributes associated with the policy tree of ciphertext to decrypt ciphertexts, and all the non-leaf nodes as necessary translation nodes. The execution times of each phase in the five schemes are shown in Figure 2, Figure 3, Figure 4 and Figure 5, respectively.

From Figure 2, the comparative analysis of the five schemes in terms of the calculation time in the setup phase. For these five schemes, the scheme of Xue et al. [31] is the longest, followed by Ours. The scheme of Bethencourt et al. [27] has the least calculation time, followed by Li et al. [15] and Zhong et al. [19], their calculation time is similar. In this phase, our scheme performs attribute encryption authentication in the initialization phase, ensuring that personal attributes are not leaked, protecting personal privacy, and higher security. The other four schemes do not have this feature.

From Figure 3, the comparative analysis of the five schemes in terms of the calculation time in the key generation phase. For these five schemes, the scheme of Li et al. [15] is the longest, followed by Zhong et al. [19]. The scheme of ours has the least calculation time, followed by Xue et al. [31] and Bethencourt et al. [27], their calculation time is similar.

From Figure 4, the comparative analysis of the five schemes in terms of the calculation time in the encryption phase. For these five schemes, the scheme of Li et al. [15] is the longest, followed by Xue et al. [31]. The scheme of ours has the least calculation time, followed by Zhong et al. [19]. The calculation time of Bethencourt et al. [27] is in the middle of the five schemes.

From Figure 5, the comparative analysis of the five schemes in terms of the calculation time in the decryption phase. For these five schemes, the scheme of Xue et al. [31] is the longest, followed by Bethencourt et al. [27]. The scheme of ours and Zhong et al. [19] has the least calculation time, their calculation time is similar. The calculation time of Li et al. [15] is in the middle of the five schemes.

## 7. Conclusions

When sharing data resources in the Internet of Things, it is of great significance to encrypt the data and set threshold functions to control the access rights of users to protect the security of the data. Information resource sharing based on the Internet of Things is highly vulnerable to external or internal threats. The complex access environment of the Internet of Things makes it easy for users’ privacy and shared resource information to be leaked or maliciously attacked. Therefore, appropriate measures must be taken to make information resource sharing more secure and reliable. This paper proposes an access control scheme based on ciphertext attribute authentication and threshold function for IoT resource sharing. Firstly, it introduces the application background and existing security challenges of the Internet of Things. Secondly, it analyzes and summarizes the relevant international research results, and introduces the research contribution of this paper. Thirdly, in order to describe the introduction of the proposed algorithm and research scheme in more detail, the main technology is to propose ciphertext identity authentication based on attribute encryption technology, which can achieve the purpose of identity authentication, and also can effectively protect the privacy of terminal members. For the information resources to be shared by the terminal members, the resource information is encrypted before being uploaded to the cloud server to prevent the terminal members who do not have the rights to access the information resources, and at the same time ensure the confidentiality and security of the data during the transmission process. When accessing data resources, set access rights to terminal members, and use attribute-based threshold functions to set access permission levels for each terminal member. Divide different permission levels according to different attributes of users, access information resources of different sensitivity levels, avoid leakage of sensitive information, and make access to information resources more flexible and quickly. Finally, the correctness and security of the proposed scheme are proved, and the computational complexity and computational time overhead of the scheme are analyzed. The proposed scheme is applicable to the resource access control of small mobile devices with limited IoT resources.

## Figures and Tables

**Table 1:** The terminal users registration process of ABE-AC.

Terminal Users		CA
$\begin{array}{c} u_{i}\left(1\leq i\leq n\right)\\ \mathrm{The}\;\mathrm{attribute}\;\mathrm{set}\;\mathrm{of}u_{i}:\\ attr_{i}=\left\{a_{i,1},a_{i,2},\ldots ,a_{i,r}\right\} \end{array}$		$\begin{array}{c} \mathrm{The}\;\mathrm{attribute}\;\mathrm{set}\;\mathrm{of}\;\mathrm{network}:\\ Attr=\{A_{1},A_{2},\ldots ,A_{j},\ldots ,A_{R}\} \end{array}$
		$\begin{array}{c} \mathrm{Construct}\;\mathrm{polynomial}\;\mathrm{function}:\\ f\left(x\right)=b_{R}x^{R}+b_{R-1}x^{R-1}+\ldots +b_{0} \end{array}$
$\begin{array}{c} \mathrm{Selects}\;a\;\;\mathrm{random}\;\mathrm{number}:\\ \lambda_{i}\in Z_{q}^{*}\left(\lambda_{i}\neq 1,0\right)\\ \mathrm{Calculates}:\\ \{\left(\lambda_{i}g_{1},a_{i,1}\lambda_{i}g_{1},\ldots ,a_{i,1}^{R}\lambda_{i}g_{1}\right),\\ (\lambda_{i}g_{1},a_{i,2}\lambda_{i}g_{1},\ldots ,a_{i,2}^{R}\lambda_{i}g_{1}),\ldots ,\\ \left(\lambda_{i}g_{1},a_{i,r}\lambda_{i}g_{1},\ldots ,a_{i,r}^{R}\lambda_{i}g_{1})\right\}\\ \beta_{i}=\left(a_{i,1}+a_{i,2}+\ldots +a_{i,r}\right)sk_{u_{i}}\lambda_{i}g_{1} \end{array}$	$\overset{\begin{array}{c} \{\left(\lambda_{i}g_{1},a_{i,1}\lambda_{i}g_{1},\ldots ,a_{i,1}^{R}\lambda_{i}g_{1}\right),\\ (\lambda_{i}g_{1},a_{i,2}\lambda_{i}g_{1},\ldots ,a_{i,2}^{R}\lambda_{i}g_{1}),\ldots ,\\ \left(\lambda_{i}g_{1},a_{i,r}\lambda_{i}g_{1},\ldots ,a_{i,r}^{R}\lambda_{i}g_{1}\right),\beta_{i},pk_{u_{i}}\} \end{array}}{⟶}$	$\begin{array}{c} \mathrm{Calculates}:\\ \gamma_{i}=a_{i,1}\lambda_{i}g_{1}+a_{i,2}\lambda_{i}g_{1}+\ldots +a_{i,r}\lambda_{i}g_{1}\\ \mathrm{Verifies}\;\mathrm{the}\;\mathrm{equation}:e\left(\beta_{i},g_{1}\right)=e\left(\gamma_{i},pk_{u_{i}}\right)\\ \mathrm{Calculates}:\\ b_{0}\lambda_{i}g_{1}+b{}_{1}a_{i,1}\lambda_{i}g_{1}+\ldots +b_{R}a_{i,1}^{R}\lambda_{i}g_{1}=\\ f\left(a_{i,1}\right)\lambda_{i}g_{1}\\ b_{0}\lambda_{i}g_{1}+b{}_{1}a_{i,2}\lambda_{i}g_{1}+\ldots +b_{R}a_{i,2}^{R}\lambda_{i}g_{1}=\\ f\left(a_{i,2}\right)\lambda_{i}g_{1},\ldots ,\\ b_{0}\lambda_{i}g_{1}+b{}_{1}a_{i,r}\lambda_{i}g_{1}+\ldots +b_{R}a_{i,r}^{R}\lambda_{i}g_{1}=\\ f\left(a_{i,r}\right)\lambda_{i}g_{1}\\ \mathrm{Computes}\;Q_{j}=A_{j}\lambda_{i}g_{1}\left(1\leq j\leq R\right)\\ \mathrm{Compares}\;A_{j}\lambda_{i}g_{1}=a_{i,\iota }\lambda_{i}g_{1}\left(1\leq \iota \leq r\right)\\ \mathrm{Chooses}\;t_{i,1},t_{i,2},\ldots ,t_{i,r}\in Z_{q}^{*}\\ \mathrm{Calculates}:\\ \{T_{i,0}=\lambda_{i}g_{1},T_{i,1}=t_{i,1}T_{i,0},T_{i,2}=t_{i,2}T_{i,0},\ldots \\ T_{i,r}=t_{i,r}T_{i,0}\}\\ \eta_{i,h}=SK_{A}\left(t_{i,1}+t_{i,2}+\ldots +t_{i,r}\right)g_{1} \end{array}$
	$\overset{\left\{\eta_{i,h},T_{i,1},T_{i,2},\ldots ,T_{i,r}\right\}}{⟵}$	
$\begin{array}{c} \mathrm{Calculates}:\\ \varepsilon_{i}=\left(t_{i,1}+t_{i,2}+\ldots +t_{i,r}\right)g_{1}\\ \mathrm{Verifies}\;\mathrm{the}\;\mathrm{identity}\;\mathrm{of}\;\mathrm{CA}:\\ e\left(\eta_{i,h},g_{1}\right)=e\left(\varepsilon_{i},PK_{A}\right)\\ \mathrm{Calculates}:\\ K_{i,1}=\lambda_{i}^{-1}T_{i,1}=t_{i,1}g_{1},\\ K_{i,2}=\lambda_{i}^{-1}T_{i,2}=t_{i,2}g_{1},\ldots ,\\ K_{i,r}=\lambda_{i}^{-1}T_{i,r}=t_{i,r}g_{1}\\ \mathrm{Obtains}:\\ \left\{K_{i,1},K_{i,2},\ldots ,K_{i,r},\eta_{i,h}\right\} \end{array}$		
	$\overset{\left\{u_{i},pk_{u_{i}},\eta_{i,h}\right\}}{\to }$	$u_{i}\left(1\leq i\leq n\right)$ register successfully

**Table 2:** The registration information of terminal users.

Terminals	*u*_1_	*u*_2_	…	*u_n_*
Effectiveness	yes	yes	…	yes
Publickey	*pk*_*u*_1__	*pk*_*u*_2__	…	*pk_u_n__*
Keywords	*keywords*_1_	*keywords*_2_	…	*keywords_n_*
Description	*D*_1_	*D*_2_	…	*D_n_*
Encryptionkey	*PK*_*g*−*u*_1__	*PK*_*g*−*u*_2__	…	*PK*_*g*−*u_n_*_
Ciphertextresource	*c*_1_	*c*_2_	…	*c_n_*
Privilege grade	*η*_1,*h*_	*η*_2,*h*_	…	*η_n,h_*
right parameter	*T*_*i*,1_,…	*T*_2,1_,…	…	*T*_*n*,1_,…
Threshold value	*y*_1,1_,…	*y*_2,1_,…	…	*y*_*n*,1_,…

**Table 3:** Symbols used mainly in this chapter.

Phase	Bethencourt et al. [27]	Xue et al. [31]	Li et al. [15]	Zhong et al. [19]	Ours
Setup	$3T_{\exp}+T_{bp}$	$\left(5+n\right)T_{\exp}+T_{bp}$	$\left(2n_{u}+4\right)T_{exp}+1T_{bp}$	$2n_{u}T_{exp}+n_{u}T_{bp}$	$2n_{u}\left|Attr\right|T_{\exp}+4T_{bp}$
KeyGen	$\left(2n_{u}+2\right)T_{\exp}$	$\left(2n_{u}+3\right)T_{\exp}$	$3n_{u}T_{exp}+n_{u}T_{bp}$	$3n_{u}T_{exp}$	$n_{u}T_{exp}$
Encrypt	$\left(2n_{c}+2\right)T_{exp}$	$(2n_{c}+3+\left|tr|\right)T_{exp}$	$\left(4n_{u}+1\right)T_{exp}+1T_{bp}$	$4T_{exp}+4T_{bp}$	$4T_{bp}$
Decrypt	$\left(2n_{c,u}+1\right)T_{bp}+(n_{c,u}+\left|nl|\right)T_{\exp}$	$(2n_{c,Attr}+|tr_{Attr}\left|+2\right)T_{bp}+(n_{c,Attr}+\left|nl|\right)T_{\exp}$	$3n_{u}T_{bp}$	$1T_{exp}+3T_{bp}$	$3T_{bp}$
